# Enzyme Catalytic Parameters and Evolution Across the Dissipation Plane

**DOI:** 10.3390/ijms27041709

**Published:** 2026-02-10

**Authors:** Davor Juretić, Branka Bruvo Mađarić

**Affiliations:** 1 Faculty of Science, University of Split, Ruđera Boškovića 33, 21000 Split, Croatia; 2 Department of Molecular Biology, Ruđer Bošković Institute, Bijenička Cesta 54, 10000 Zagreb, Croatia; branka.bruvo.madjaric@irb.hr

**Keywords:** evolution, enzyme efficiency, turnover number, evolutionary distances, partial entropy production, thermodynamic constraints, scale-invariant dissipation plane

## Abstract

Enzyme performance parameters, including the turnover number and specificity constant, exhibit remarkable diversity due to biological evolution and natural selection. In some bacterial and human enzymes, catalytic efficiencies approach fundamental physical limits, underscoring the importance of physical constraints on enzymatic function. A deeper understanding of these constraints, particularly in far-from-equilibrium irreversible processes, is therefore essential for rational enzyme engineering. Such constraints are most naturally addressed within the frameworks of nanothermodynamics and stochastic thermodynamics, which remain relatively unfamiliar to much of the molecular biology community. Recent theoretical and experimental advances indicate that classical enzyme kinetic parameters are not independent, but are systematically linked to energetic dissipation. In particular, enzymes appear to occupy a characteristic dissipation plane defined by entropy production, reflecting the coupled influence of thermodynamic principles and evolutionary selection. In this review, we synthesize evidence across diverse enzyme families demonstrating correlated increases in housekeeping dissipation, evolutionary divergence, and enzymatic performance. Together, these findings support dissipation as a physically grounded parameter that connects enzyme kinetics, biological evolution, and nonequilibrium thermodynamics.

## 1. Introduction

Although there is no universal scientific consensus on origin and a precise definition of life [1,2], modern biology converges on measurable hallmarks such as metabolism, growth, and energy dissipation. Among these, metabolic activity provides a quantifiable physical signature of living systems. Vigorously reproducing bacterial or yeast cells acts as a measurable heat source. Exported dissipation is also associated with the evolutionary emergence and maturation of complex heterotrophs, endotherms, and ecological systems [3,4,5]. Thus, the additional heat dissipated into the environment indicates the presence of metabolism in living cells [6].

### 1.1. Enzymatic Catalysis

Interest in the relationship between life and entropy production due to metabolism has increased markedly over the past decade. This viewpoint from physics is steadily entering mainstream thinking about the origin and evolutionary divergence of molecular biology [7,8,9,10,11,12,13]. However, metabolism and molecular biology ultimately rely on highly evolved enzymes that accelerate chemical reactions by extraordinary amounts [14,15]. At the molecular level, enzymes act as the primary mediators through which biological systems harvest and channel available free energy into directed, dissipative processes.

Understanding enzymatic catalysis conventionally emphasizes the roles of structure and electrostatics [16], with dynamics entering through the mobility of functionally important residues, conformational substates, hydride transfer, and loop motions [17,18,19]; together, these factors determine enzyme kinetic and thermodynamic parameters. Predicting such kinetic parameters has become an active research direction [20,21,22,23,24]. In contrast, identifying statistically meaningful relationships between enzyme kinetic parameters and dissipation caused by evolved or designed enzymes remains comparatively underexplored. This review focuses on the relationship between enzyme kinetic parameters and entropy production during catalysis, examining theoretical frameworks, experimental evidence, and computational approaches, with particular emphasis on how evolutionary pressures may have shaped these properties.

### 1.2. Enzyme Evolution

Enzyme evolution has been extensively studied in molecular biology and biochemistry, giving rise to a large and mature literature that emphasizes genetic variation, protein structure and dynamics, catalytic mechanisms, organismal fitness, and directed evolution through enzyme engineering [25,26,27]. Over the past decade alone, this mainstream framework has been summarized in several hundred review articles, reflecting the depth and diversity of established approaches to understanding the origin, diversification, and optimization of enzymatic function (e.g., reviews focusing on gene duplication and divergence, enzyme promiscuity and specialization, and structure–function relationships) [28]. Representative examples of these perspectives are briefly outlined here, and readers are directed to recent, high-quality review articles that treat these topics in depth [25,27]. The present review, therefore, does not aim to comprehensively survey or critically compare the full range of conventional evolutionary models, but instead focuses on an emerging perspective: how nonequilibrium thermodynamic constraints, expressed through dissipation and entropy production, may restrict enzyme catalytic parameters and thereby influence enzyme evolution, whether natural or engineered.

### 1.3. Intertwined Physical and Biological Evolution

High dissipation characterizes far-from-equilibrium processes. While such processes are not unique to living systems, their sustained and regulated manifestation through metabolism is a defining feature of life [29]. When associated with inanimate systems, driven irreversible processes direct physical evolution; analogous principles are expected to operate in living systems. Biological systems, therefore, operate under universal thermodynamic constraints, with evolutionary selection acting on molecular mechanisms—particularly enzymes—that exploit these constraints. Consequently, physical and biological evolution are intimately linked in shaping enzyme function [30].

This line of reasoning raises the question of how intertwined physical and biological evolution have left their imprint on enzymes. In this review, we analyze whether commonly measured enzyme kinetic parameters—such as turnover numbers and catalytic efficiencies—exhibit systematic relationships with entropy production during catalysis. By integrating perspectives from enzymology, nonequilibrium thermodynamics, and evolutionary biology, we assess whether the dissipation landscape represents an emergent, population-level design principle of evolved enzymes.

## 2. Tools Choice from Irreversible Thermodynamics

### 2.1. Prigogine’s Approach and Stochastic Thermodynamics

Let us use the symbol σ for local entropy production. For isothermal conditions, the dissipation function Φ is proportional to *σ*: Φ=T·σ, where *T* is the absolute temperature. Entropy production measures the irreversibility of a process, while the dissipation *T* · *σ* measures the energy cost of that irreversibility, i.e., how much free energy is lost as heat. In living cells or organisms, enzymes operate in an open, energy-driven system far from thermodynamic equilibrium. The system can exchange energy (heat, light, chemical work), matter (nutrients, products, ions), and information (signaling, regulation) with its environment. Irreversible thermodynamics is an appropriate tool to study living systems. When we focus on enzymes, the question becomes: should we use modern stochastic thermodynamics for microscopic irreversible processes [7,31] or the older Prigogine-like description [32,33], which is suitable for driven open systems? However, the definition of entropy production differs between the modern and coarse-grained approaches.

Stochastic thermodynamics treats the system and the heat bath as a closed universe. It then defines the entropy production as the sum of the system term and bath entropy change [31]. Initial requirements of no matter flow across the system boundary [7,34] were subsequently relaxed [35,36,37,38,39]. The constant-temperature requirement was also relaxed [36,40,41]. However, the assumption of a closed, energy-conserving “universe” needed to derive stochastic thermodynamics from the microdynamics of a closed total system remained the best way to consistently define entropy production microscopically. Some ingenious exceptions were found on how to go around that obstacle, too. For instance, one can add an information reservoir to the system and a heat bath to achieve a closed total system [42,43]. Also, for active matter and self-driven systems, the coarse-grained effective baths can be added to restore the ability to calculate the entropy production rates [44,45,46,47]. The “environment” is then effectively open due to the postulated existence of a large thermostat that is not explicitly included.

Over the last two decades, stochastic thermodynamics has been applied to model biomolecules in vitro, such as a motor protein in a well-defined chemical bath (ATP/ADP + buffer) [48] or a single attached enzyme [49,50]. Nevertheless, it is not the simplest or best framework for modeling a living cell or enzymes coupled to multiple reservoirs (chemical, mechanical, electrical, heat, radiation), which can exchange energy and matter with their surroundings. Those systems are open and far from equilibrium, where energy fluxes through the system maintain order and drive metabolism—exactly the domain of Prigogine’s approach.

**Figure 1:**
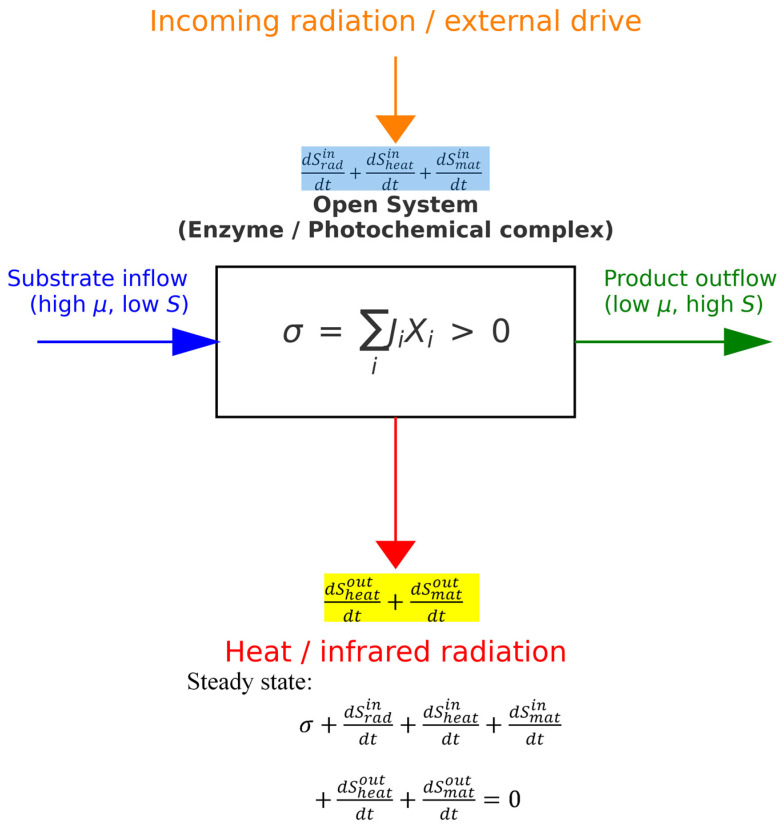
A general Prigogine-like scheme for an open steady-state system performing irreversible free-energy transduction [32,33]. All generalized forces *X_i_* and corresponding flows *J_i_* (radiation, heat, matter) contribute to the entropy production density σ, expressed as the sum of *J_i_X_i_* products. In particular, enzymes or living systems convert high free-energy *μ* and low-entropy *S* substrates into low free-energy and high-entropy products.

The central square of Figure 1 represents an open system. Given multiple inputs and outputs (arrows), one can choose the conditions for the system of interest. For short time periods, we can approximate the homeostasis of the living system as a steady-state condition, in which there is no change in entropy, temperature, or pressure. The radiation field can also be excluded when the proteins of interest do not include light detectors, light converters, or photosystem complexes. That simplifies the measurement of the entropy produced within the system (due to irreversible processes), which is completely exported to maintain the steady-state zero entropy change for the system. Microcalorimetric techniques [51,52,53] are used to achieve that goal.

### 2.2. Decomposition of Entropy Production

The decomposition of entropy production *σ* into the sum of forces *X_i_* and corresponding flows *J_i_* (as depicted in Equation (1) and the central square of Figure 1) is the essential relation of irreversible thermodynamics. The bilinear form *ΣJ_i_X_i_* is a consequence of the second law of thermodynamics and the local equilibrium assumption [54]. At fixed thermodynamic forces and positive *σ*(*J_i_*) = *ΣJ_i_X_i_*, only a maximum can be the extremum of σ (maximum entropy production principle) [54]. The principle enables the calculation of actual (optimal) thermodynamic flows. In 2017, Martyushev and Celezneff [55] showed that replacing the assumption of local equilibrium with the postulate of scale invariance *σ* = *σ*(*J*) allows one to construct nonequilibrium thermodynamics that not only includes all the results of classical linear nonequilibrium thermodynamics, but also allows one to describe nonlinear systems that are extremely far from equilibrium. Very few exceptions have been claimed regarding the *ΣJ_i_X_i_* bilinearity in non-equilibrium thermodynamics [56]. Other authors re-examined these exceptions (see Section 4).

(1) $$\sigma =\sum_{i}J_{i}X_{i}$$

Forces and flows are generalized quantities. Enzyme biochemistry considers chemical affinities as forces and corresponding conversions of substrates into products as flows. Equation (1) is the theoretical way to calculate entropy production when we can measure or calculate all flow-force couples relevant to the biochemical pathway of interest. We do not have to worry about how entropy is defined, whether we are close or arbitrarily far from thermodynamic equilibrium, nor whether the relationship between flows and forces is linear or nonlinear. For enzyme catalyzed reactions $\sigma =\sum_{i}J_{i}\frac{A_{i}}{T}$, where $A_{i}$ is the affinity for each reaction *i* at the temperature *T*. While the dissipation is due to internal irreversible processes (flows) according to Prigogine’s definition, forces can be either internal or external. External forces are treated as boundary conditions.

When averaged, the microscopic definition of entropy production from stochastic thermodynamics also reduces to the Equation (1) definition [57,58]. Nevertheless, Prigogine’s definition has been applied to small-system nanothermodynamics in biochemistry and stochastic thermodynamics by Terrel L. Hill and other researchers [36,59,60,61,62,63,64,65,66]. Tanford (1982) [67] offered the critique of Hill’s bookkeeping for free-energy transduction, but modern developments tend to support Hill’s viewpoint [68,69,70].

In Hill’s approach, one can start from measured and estimated microscopic rate constants to calculate the probability of enzyme states as the enzyme cycles among conformations and excited states during the transformation of substrates into products. Our attention is then focused on the functionally essential conformational states of the enzyme of interest, rather than on small organic molecules serving as substrates or products. From steady-state probabilities, all fluxes and forces can be easily calculated using the diagram method (appropriate for simpler kinetic schemes) or computational tools (for more complex schemes). Terrel L. Hill used toy models to illustrate how such calculations can determine the efficiency of free-energy transduction in the steady state. He explicitly stated that light-driven systems are not amenable to such an approach [59].

## 3. Entropy Production Can Be Decomposed into Productive and Waste Parts

The idea that entropy production can also be decomposed into “productive” (useful, flux-carrying, ligand-transforming, or energy-transducing) and “futile” (waste, thermal, or dissipative slip) parts merits short elaboration because readers may not be familiar with it. Also, some renowned scientists who have made fundamental contributions to nonequilibrium thermodynamics are strongly opposed to focusing on entropy production or dissipation. To cite Professor R. Dean Astumian [71]: “The focus on dissipation is misguided. Dissipation is waste and drives nothing other than heating.” However, classical network thermodynamics and stochastic thermodynamics often examine cycle decomposition of entropy production. The theory can be applied to enzyme-controlled processes. Enzyme catalysis is necessarily cyclical in nature because substrates are altered, but the enzyme itself always returns to its original state. For instance, productive and futile pathways for cycle-associated free-energy transduction and entropy production contributions were studied in detail by Terrel L. Hill [59,60,72,73] and further developed by Jürgen Schnakenberg [74] and other researchers [75,76,77,78,79].

Interestingly, both productive and futile cycles are subject to regulation and feedback [80,81,82,83,84,85,86]. For instance, the productive ATP synthesis/hydrolysis cycle is actively switched, inhibited, or modulated, which directly regulates both output and dissipation [87,88,89]. The rate and dissipation of the productive ATP-generating cycle are controlled by energy demand (ADP, ATP, NADH levels, membrane potential, and pH gradient across membranes) [90,91,92]. The productive pumping cycle of Na^+^/K^+^-ATPase (which moves Na^+^ and K^+^ across the membrane via ATP hydrolysis) is regulated by accessory proteins and phosphorylation, thereby controlling both flux and energetic cost [93,94]. For regulated dissipation in the productive cycle of molecular motor proteins, kinesin and myosin are good examples of how load and gating alter cycle kinetics, partitioning ATP energy between productive motion and futile/regulatory internal transitions [48].

## 4. Extensions of Terrell Hill’s Theoretical Approach

Meszéna and Westerhoff examined whether photosynthesis can be described as any other biochemical process [95]. They found expressions for the non-zero photon chemical potential and the net rate of absorption. By showing that photosynthetic and photochemical processes can be expressed using a generalized free energy for photons, they enabled a thermodynamically rigorous description of light-driven biochemical cycles. Their expressions for fluxes and forces associated with the radiation field can be incorporated into the bilinear form (1) to calculate the entropy production rate (EPR). When thermal dissipation of the excited state is included, we have the light-driving cycle that enables photosynthesis [96]. Juretić and Županović extended the framework to irreversible thermodynamics with radiation fields for the case of bacterial photosynthesis, treating photons analogously to reactants with an effective chemical potential/affinity [97,98]. As a result, the entropy production retains a bilinear form even when the radiation fields are far from equilibrium. Other authors also increased the scope of nonequilibrium thermodynamics of chemical reaction networks to include incoherent light as a source of free energy by defining a chemical potential of photons under local-equilibrium assumptions, thereby allowing photon fluxes and affinities to be introduced [99]. Landi and Paternostro provided a modern theoretical background for how entropy production expressions can be generalized in quantum and classical systems (including radiation) beyond the classical flux–force linear regime [100].

Our other extension of Hill’s theoretical approach is the possibility to find maximal partial entropy production (MPEP) for a chosen *i* → *j* transition [2,97,101,102]. The MPEP conform with the general formulation of the maximum entropy production principle (MEPP) [103,104] that can be formulated as “at each level of description, with preset external constraints, the local relationship between the cause and the response of a complex nonequilibrium system is established in order to maximize the entropy production.” The maximum in a chosen transition arises from a simple trade-off between thermodynamic flux and force when we vary only the forward microscopic rate constant *k_ij_*. There were no restrictions on network complexity, the nonlinearity of the force-flux response, or the distance from the equilibrium state. Considering the transitions between macromolecular steady states provides a more detailed decomposition of overall entropy production than the cycle-associated decomposition we considered in Section 3. Intriguing results are facilitated rate-limiting transitions when optimal *k_ij_* values are close to the observed values [2,97,101,102,105,106]. Such transitions in productive, free-energy transducing cycles are, as a rule, the latest and slowest recovery steps, involving obligatory proton shuffling or transport between conformations or compartments. The examples we studied in the 2003–2021 period extended physical applications of MEPP [54,107,108,109,110,111,112,113] to enzyme kinetics.

In applying these advances, we always strived to compare theoretical predictions with experimental observations. Our goal was to determine whether the observed kinetic parameters are close to the predicted optimal kinetic parameters for specific biochemical systems. Emerging insights allowed us to postulate the evolution-coupling hypothesis—biological evolution does not act independently from the physical (thermodynamic) evolution [30,114]. Evolution in biology is a broader concept than classical Darwinian evolution [115,116,117,118], but the recognition of universal physical evolution (thermodynamic evolution) is the essential achievement in physics [119] that gained only a sparse foothold in modern biology [120,121]. There is an astonishing opinion difference about the thermodynamic evolution concept among experts from different research fields.

Although the term *thermodynamic evolution* is not widely used within enzyme evolution research per se, thermodynamic principles play a central role in many physical and natural systems across disciplines. In non-equilibrium thermodynamics, the *entropy production rate* is a fundamental measure of how systems evolve over time under gradients of temperature, concentration, or chemical potential, governing processes from chemical reactions to fluid flows and plasma dynamics. For instance, in materials science and crystallization, the rate of entropy production per unit area can govern morphological pattern formation as a system moves away from equilibrium, influencing dendritic growth and surface instabilities [122]. In thermodynamic modeling of complex systems and machine performance, changes in entropy production are directly linked to energy dissipation and conversion efficiency, such as in heat engines or reactive flows studied in fluid mechanics and process engineering [123,124]. Similarly, in astrophysics, the thermodynamic properties of large-scale phenomena, such as *coronal mass ejections*, evolve with density, temperature, and pressure as the system propagates outward from the sun, reflecting changes in internal entropy and energy distribution during expansion [125]. Moreover, recent interdisciplinary work has begun to apply “thermodynamic evolution” concepts to biological systems, proposing that higher entropy production is associated with greater dissipation and may correlate with improved efficiency or complexity in biological catalysts such as enzymes. In these kinetic models, more specialized enzymes tend to dissipate more free energy, suggesting a thermodynamically driven component to their evolutionary trajectory [30]. Thus, while researchers focused on enzyme evolution or molecular biology may not routinely frame their work in terms of *thermodynamic evolution*, non-equilibrium thermodynamics and entropy production provide a unifying language that connects physical pattern formation, energy dissipation, and the behavior of complex systems across scales—from crystals and plasmas to evolving biological molecules. There is a firm feedback between biological and thermodynamic evolution. Despite being constrained by physical laws, biological evolution can accelerate or slow thermodynamic evolution [2,30].

## 5. Examples of Applications Combining Nanothermodynamics and Entropy Production Principles in Bioenergetics

### 5.1. Maximum Entropy Production Principle and Photosynthetic Models

Firstly, we examined simplified photosynthetic models of bacterial photosynthesis and bacteriorhodopsin regarding the initial irreversible photosynthetic steps [97]. In both models, free energy transduction in the productive cycle converts photon-free energy into the proton-motive force. Experimentally determined kinetic constants were published by van Rotterdam in his PhD thesis [126] for the initial photosynthetic steps of the purple photosynthetic Eubacterium *Rhodobacter sphaeroides*. For our work, we simplified his kinetic scheme to the three-state and five-state models, retaining only the essential kinetic steps of the chlorophyll-based photosynthetic cycle. In an iterative self-consistent procedure, we simultaneously sought the maximum partial entropy production in two final transitions from the productive cycle leading to active proton transport and the recovery of the bacteriochlorophyll ground state [2,97,114].

At that time, we did not have proof that the maximum in the partial edge-wise entropy production can always be found. Nevertheless, we claimed that we applied the maximum entropy production principle (MEPP). Despite the model’s simplicity and theoretical shortcomings, several interesting results followed. The dominant contribution to total entropy production of 80 to 90% is the sum of partial entropy productions in the productive (non-slip) pathway. For different choices of light intensity and the radiationless rate constant to the ground state, we obtained optimal quantum yields ranging from 0.93 to 0.98, in accord with experiments. The optimized model reproduced the backpressure regulation of energy transduction, namely that proton pumping slows with increasing transmembrane potential [127]. The microscopic recovery rate constant is rate-limiting. Its predicted optimal value, *k_recovery_* = 10.1 s^−1^, was in good agreement with the observed value, *k_recovery_* = 8.6 s^−1^ [126]. An optimal value of free-energy transduction efficiency in the range of 17–19% and a close-to-80% loss of photon input power as heat also agrees with experimental observations [2].

The other example we studied is the bacteriorhodopsin from *Halobacterium salinarium*. It is the simplest and smallest light-activated proton pump that nature designed [128]. The MPEP application yielded an optimal recovery *k_ij_* that was similar to the observed ground-state restoration rate constant. Also, the predicted hierarchy of characteristic time values was in rough agreement with the observed values. Interestingly, these results were robust with respect to the kinetic scheme we used, the number of functional states, and the presence or absence of a slip transition [2,97,98,129]. Relatively low thermodynamic efficiency in converting photon-free energy into the electrochemical proton gradient (10–16%) begs the question of why nature disregards the majority of available energy. We can assume that fundamental physical and functional (biological) characteristics of energy conversion have become tightly regulated and optimized during biological evolution. If this is so, we expect that MPEP optimization (with all constraints taken into account) cannot change the already optimal values of these parameters inferred from measurements by much. Computational experiments confirmed these expectations [102,106,129]. Biological evolution used almost all available options to accelerate enzyme evolution toward more efficient dissipation of free energy gradients, often with a coincidental increase in catalytic performance as a positive side effect. Indeed, only small increases in entropy production, net proton flux, and energy conversion efficiency can be achieved after MPEP optimization in the last (recovery) step of the bacteriorhodopsin photocycle [2,102].

The distribution of microscopic kinetic constant values over many orders of magnitude is the well-known and frequently observed feature of light-activated biological cycles. It starts with initial picosecond transitions and proceeds to the final (recovery) millisecond step [130,131,132]. That result follows naturally from our optimization method for maximal partial entropy productions [2,102], but, as far as we can tell, it has not been reproduced using the same or different extremal principles. Nothing similar could be obtained after the application of Prigogine’s minimum entropy production theorem [32].

### 5.2. Housekeeping Contribution of Edge-Wise Entropy Productions

Dhanuka et al. [133] developed an ambitious framework of ecosystems as adaptive living circuits. Local dissipation along the chosen circuit edge can be tuned to reach a near-maximal value when subjected to sufficient drive. Their “local dissipation” definition needs semantic clarification, as other authors have used different names for the same mathematical entity. We settled on the phrase “partial entropy production” [2,30,101,134] for the equivalent definition from the thermodynamic network theory, but not before using other phrases, such as “entropy production in each transition between neighboring states” [97], “free-energy dissipation associated with corresponding transition” [114], and “transitional entropy production” [102,129]. The mathematical equivalence is apparent when the *ij* edge energy dissipation [133]:

(2) $$\sigma_{ij}=\left(W_{ij}p_{j}-W_{ji}p_{i}\right)log\frac{W_{ij}p_{j}}{W_{ji}p_{i}}$$

is compared to the partial dissipation for the net mean transition flux *i* → *j* [55,98]:

(3) $$\sigma_{ij}T=k_{B}T\left(k_{ij}p_{i}-k_{ji}p_{j}\right)log\frac{k_{ij}p_{i}}{k_{ji}p_{j}}$$

where *k_B_* is the Boltzmann constant, *T* is temperature, *p_i_* are state probabilities, *k_ij_* are microscopic kinetic constants, and the number of moles *n* is taken to be one. The physical and biological context is very different. In our case, we do not deal with living species as edges of the circuits, but with macromolecules (enzymes), their functionally important conformational states, and their interstate transitions, which may involve smaller molecules (substrates, products) with which they interact. The decomposition of total entropy production (or dissipation) into positive partial contributions is a generally accepted procedure in irreversible thermodynamics and bioenergetics [2].

Still, two clarifications would help with the phrase “partial entropy production”. Firstly, the term “partial entropy production” is used in recent literature to indicate *incomplete knowledge* [135] rather than a desired positive additive partition of a fully known total. Secondly, recent decomposition proposals of entropy production for general nonlinear dynamics distinguish the housekeeping cycling mode (due to external driving) and the excess part originating from the relaxation mode [136]. The housekeeping positive contribution is the entropy production part, which can be considered productive, as we discussed in Section 3, but with different mathematical and physical meanings from those used in our biochemical network models. However, Yoshimura et al. [136] also provided the mathematical decomposition of housekeeping contribution into the sum of positive *J_e_X_e_* products, where *X_e_* is the thermodynamic force on edge *e*, while *J_e_* is the current on edge *e*. In a steady-state situation, when each node in a biochemical kinetic scheme represents the conformation of an enzyme or an enzyme complexed with small molecules, and each edge represents a bidirectional transition between conformations, this is equivalent to the EPR decomposition used by Terrel L. Hill [59] (see his Equation 4.28) and us (our publications [2,30,97,101,102,105,106,129,134] in the 2003–2025 period). Thus, the essential caveat about our usage of the “partial entropy production” phrase is its connection to the edge-wise or transition-wise contribution to the known total EPR.

For instance, when the transition-state parameter *κ* (defining the angular position for the transition state) is varied in modeling F_1_-ATPase, a single joint maximum is found for the information entropy and the entropy production in the transition responsible for ATP synthesis or hydrolysis [101]. In the intact F_0_F_1_-ATP-synthase, ATP synthesis or hydrolysis is coupled to transmembrane proton translocation. After MPEP application, the optimal *κ_optimal_* = 0.602 for chloroplast’s ATPase turned out to be very close to observed *κ_observed_* = 0.598 [137]. The transition state theory and our modeling reproduced the sigmoidal shape of the ATP synthesis flux observed by Pänke and Rumberg [137]. Sigmoidal enzyme kinetics is well known as a sign of cooperativity and metabolic regulation [138,139,140,141]. The inflection point of a sigmoidal reaction rate curve is the point of maximal sensitivity to variable chemical potential when the ATP synthesis rate is maximally sensitive to the electrochemical proton gradient. We found that the inflection point is also (a) the point of maximal information entropy, (b) the point of maximal partial entropy production, and (c) the point of the best far-from-equilibrium linearity in the force-flux relationship. Thus, we claimed that the optimal metabolic control is achieved for maximal partial entropy production [101]. Subsequently, metabolic regulation was linked to various versions of the maximal entropy production principle [98,105,142,143,144,145,146,147,148,149,150,151].

In conclusion, we addressed in this chapter the fundamental question linking bioenergetics and nonequilibrium physics: can the evolved dissipative steps that facilitate biomolecular function be identified by their contribution to the housekeeping entropy production? We have found that dominant entropy-production transitions are coupled to nonequilibrium conformational switches that enable ATP synthesis or the active transport of protons. Such localized or edge-wise transitions can be optimized by using the MPEP theorem [102]. Optimized parameters are often close to the observed values in accord with MEPP [104].

### 5.3. Thermodynamic and Kinetic Parameters for Single-Cycle Enzymes

We have also recently examined simpler one-cycle enzymes that maintain nonequilibrium concentrations of biologically important small molecules without free-energy transduction [30,134,152]. A sufficiently large dataset of enzymes with known microscopic rate constants enables not only the calculation of *k_cat_*, *K_M_*, and overall entropy production for individual enzymes, but also statistically meaningful analyses of the interdependence between biochemical and physical parameters. In two recent studies [30,134], we restricted the dataset to enzymes operating through a single productive catalytic cycle, characterized by a single steady-state flux passing through two, three, or four enzyme conformational states, with or without bound ligands. All analyses were performed under steady-state conditions [59].

To facilitate reading, we reproduce here (from [30]) the equations we used to calculate *k_cat_*, *K_M_*, *k_cat_*/*K_M_*, and overall entropy production from microscopic rate constants. We assumed reversible (bidirectional) transitions among functionally important enzyme conformations E (1), ES (2), EP (3) or EZ (3), and EP (3 or 4) and predominantly counterclockwise cycling among states (see reversible kinetic schemes contained within Figure 1 from [30]). All transitions between enzyme conformational states are treated as first-order processes. For example, for the binding transition E + S → ES, we use $k_{1}=\;k_{1}^{*}[S]$, where $k_{1}$ is the first order rate constant (in s^−1^ units), $k_{1}^{*}$ the second-order rate constant (in M^−1^s^−1^ units), and [*S*] is the concentration of substrate (in moles). The same notation is used for product-binding transitions. Forward kinetic constants are *k*_1_, *k*_3_, *k*_5_, and *k*_7_. Reverse kinetic constants are *k*_2_, *k*_4_, *k*_6_, and *k*_8_. For the two-state reversible cycle:

(4) $$k_{cat}=k_{3}\;\;\;\;\;\;\;\;\;\mathrm{and}\;\;\;\;\;\;\;\;\;\frac{k_{cat}}{K_{M}}=\frac{k_{1}k_{3}}{\left[S\right](k_{2}+k_{3})}$$

For the three-state cyclic kinetic mechanism:

(5) $$k_{cat}=\frac{k_{5}}{1+\frac{k_{4}}{k_{3}}+\frac{k_{5}}{k_{3}}}\;\;\;\;\;\;\;\;\;\mathrm{and}\;\;\;\;\;\;\;\;\;\frac{k_{cat}}{K_{M}}=\frac{k_{1}k_{3}k_{5}}{\left[S\right](k_{2}k_{4}+k_{2}k_{5}+k_{3}k_{5})}$$

For the four-state cyclic kinetic mechanism:

(6) $${\;\;k}_{cat}=\frac{k_{3}}{1+\frac{k_{3}}{k_{7}}+\frac{k_{3}}{k_{5}}\left(1+\frac{1}{K_{2}}\right)\left(1+\frac{1}{K_{3}}\frac{k_{5}}{k_{7}}\right)}$$

and

(7) $$\frac{k_{cat}}{K_{M}}=\frac{{k_{1}k_{3}k_{5}k}_{7}}{\left[S\right](k_{2}k_{4}k_{6}+k_{2}k_{4}k_{7}+k_{2}k_{5}k_{7}+k_{3}k_{5}k_{7})}$$

The two-state expressions for net reaction flux *J* and the thermodynamic force *X* are, respectively

(8) $$J=\frac{k_{1}k_{3}-k_{2}k_{4}}{k_{1}+k_{2}{+k}_{3}+k_{4}\;\;\;}\;\;\;\;\;\;\;\;\;\mathrm{and}\;\;\;\;\;\;\;\;\;X=RTln\frac{k_{1}k_{3}}{k_{2}k_{4}}=RTlnK$$

where *K* = *K*_1_ ∙ *K*_2_ is the equilibrium constant. Three-state expressions are:

(9) $$J=\frac{k_{1}k_{3}k_{5}-k_{2}k_{4}k_{6}}{k_{1}\left({k_{3}+k_{4}+k}_{5}\right)+k_{2}k_{4}+k_{2}k_{5}{+k}_{3}k_{5}+k_{6}\left({k_{2}+k_{3}+k}_{4}\right)}$$

and

(10) $$X=RTln\frac{k_{1}k_{3}k_{5}}{k_{2}k_{4}k_{6}}=RTlnK$$

with *K* = *K*_1_ ∙ *K*_2_ ∙ *K*_3_.

Four-state expressions are:

(11) $$J=\frac{k_{1}k_{3}k_{5}k_{7}-k_{2}k_{4}k_{6}k_{8}}{\Sigma_{1}+\Sigma_{2}+\Sigma_{3}+\Sigma_{4}}$$

with

$$\Sigma_{1}=k_{2}k_{4}k_{6}+k_{2}k_{4}k_{7}+k_{2}k_{5}k_{7}+k_{3}k_{5}k_{7}\;\Sigma_{2}=k_{1}k_{5}k_{7}+k_{4}k_{6}k_{8}+k_{1}k_{4}k_{6}+k_{1}k_{4}k_{7}\;\Sigma_{3}=k_{1}k_{3}k_{7}+k_{2}k_{6}k_{8}+k_{3}k_{6}k_{8}+k_{1}k_{3}k_{6}\;\Sigma_{4}=k_{2}k_{4}k_{8}+k_{1}k_{3}k_{5}+k_{3}k_{5}k_{7}+k_{2}k_{5}k_{7}$$

and

(12) $$X=RTln\frac{k_{1}k_{3}k_{5}k_{7}}{k_{2}k_{4}k_{6}k_{8}}=RTlnK$$

where *K* = *K*_1_ ∙ *K*_2_ ∙ *K*_3_ ∙ *K*_4_.

The dissipation function is then the *J* ∙ *X* product in each case.

The question we asked was whether there is a statistically significant relationship between biochemical (kinetic constants) and physical performance parameters (housekeeping entropy production). It turned out that enzyme kinetic parameters, such as the turnover number *k_cat_* and the Michaelis–Menten constant *K_M_*, are not independent of each other or of the total entropy produced. The specificity constant, as the ratio of these two parameters (*k_cat_*/*K_M_*), is proportional to the dissipated energy in the log-log frame.

It is worthwhile to note that determining *k_cat_* and *K_M_* is often regarded as a complete characterization of any enzyme-catalyzed reaction [53]. However, the connection between biochemical and physical parameters is less well known. It survived the eons of biological evolution and exhibits a clear trend: more evolved members of the same enzyme family are associated with greater specificity toward their substrate and higher entropy production [2,134]. By examining a large enough set of well-known enzyme families, we discovered yet another general rule—specialized enzymes are considerably better dissipators from bi-functional and generalist enzymes, thus confirming Jensen’s hypothesis (that specialized enzymes emerged from generalized enzymes during biological evolution) [153,154] in an unexpected way [134].

## 6. The Dissipation-Scaling Plane

### 6.1. Power-Law Scaling Between Dissipation and Enzyme Kinetic Parameters

For the selected 75 enzymes, their overall steady-state dissipation and kinetic parameters can be easily calculated when all microscopic rate constants are known in both forward and backward directions [30,59,134] due to their simple, reversible catalytic schemes. The power-law scaling exists between dissipation and enzyme efficiency *k_cat_*/*K_M_* and also between dissipation and *k_cat_* [134]:

$$\frac{dissipation}{RT}=10^{a1}\cdot {\left\{\frac{k_{cat}}{K_{M}}\right\}}^{b1}\;\;\;\;\;\;\;\;\;\mathrm{and}\;\;\;\;\;\;\;\;\;\frac{dissipation}{RT}=10^{a2}\cdot {\left\{k_{cat}\right\}}^{b2}$$

After taking the logarithm, a high degree of fit and significant power-law scaling is found [134]. The exponent (slope) *b*1 = 0.72, while *b*2 = 0.97. The difference between the slopes is statistically significant, as indicated by a narrow, robust confidence interval (small standard errors for both slopes).

**Figure 2:**
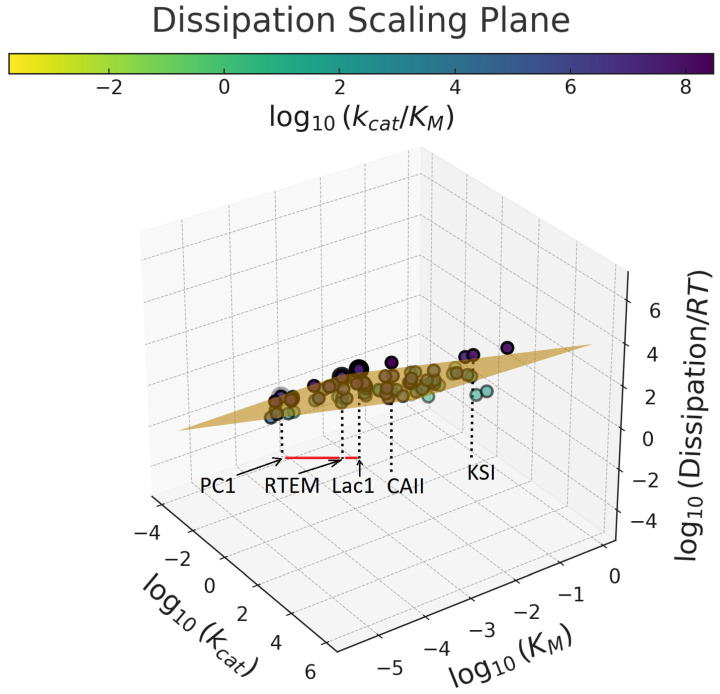
The dissipation plane viewpoint of our recent results [134]. Seventy-five points for 75 enzyme-catalyzed reactions are mostly contained within the brown plane in such a 3D log-log-log representation that the *x*-axis is log_10_(*k_cat_*), the *y*-axis is log_10_(*K_M_*), and the *z*-axis is log_10_(Dissipation/(*RT*)). Points are colored by the specificity constant (*k_cat_/K_M_*), also named enzyme efficiency. The vertical projections to the (*x*,*y*) plane (dotted lines) highlight five reactions associated with an increased overall dissipation, ranked as CAII (carbonic anhydrase II) > KSI (ketosteroid isomerase) > Lac1 > RTEM > PC1. The last three enzymes are evolutionarily related β-lactamases from bacteria, likely descended from the same ancestor. That is why we highlighted them with black arrows and red connecting lines.

Figure 2 presents a three-dimensional log–log–log representation of these results for the same 75 enzymes. Although this visualization does not introduce new data, it provides an alternative perspective on our recent two-dimensional analyses [134]. The 3D plot highlights the existence of a dissipation-scaling plane that accommodates enzymes spanning the full spectrum of catalytic performance, from sluggish enzymes (yellow and green points) to those approaching catalytic perfection (dark pink points). Notably, 20 of the 75 reactions are catalyzed by artificially mutated enzymes. Their inclusion enables partial disentanglement of purely physical constraints imposed by protein structure from biologically evolved structure–function relationships. Remarkably, engineered enzymes populate the same dissipation plane as their naturally evolved counterparts, with nearly identical goodness-of-fit values and statistically indistinguishable scaling slopes [134]. This observation demonstrates that the dissipation–specificity scaling relationship persists even in the absence of evolutionary history, underscoring its physical origin and generality. Figure 2 further reveals that enzymes with near-optimal catalytic performance are associated with exceptionally high dissipation. Among these are three bacterial β-lactamases with corrected Ambler sequences [102,155,156,157]: *Staphylococcus aureus* PC1, *Escherichia coli* RTEM, and *Bacillus cereus* Lac1. The red connecting lines illustrate their evolutionary relationship, which is accompanied by concomitant increases in both the catalytic constant *k_cat_* and overall dissipation.

### 6.2. Dissipation, Performance, and Phylogenetic Analysis

**Figure 3:**
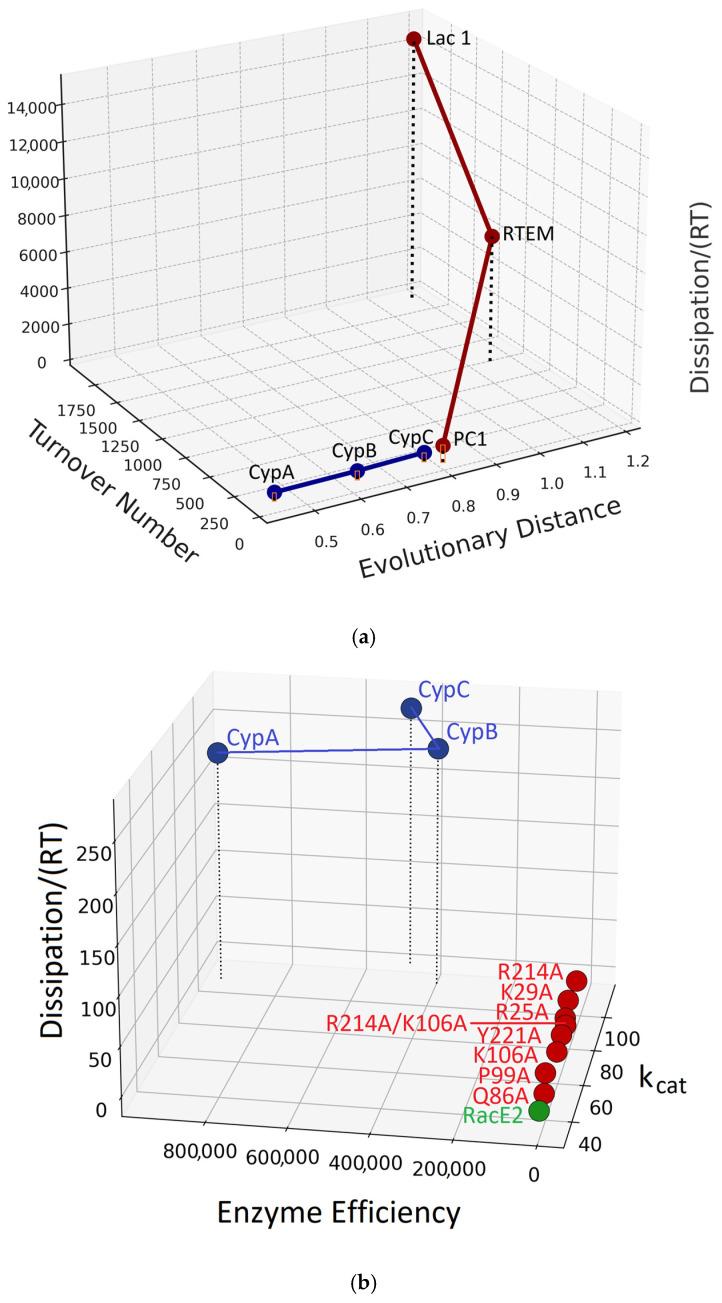
(**a**) The connection of biochemical, evolutionary, and physical parameters for cyclophilins (blue points and connecting lines) and β-lactamases (red points and connecting lines). The methods already described [134] were applied to find evolutionary distances. There was no change in cyclophilin distances, and we reproduced the ranking of evolutionary distances for bacterial β-lactamases: PC1 = 0.8119 < RTEM = 1.1456 < Lac1 = 1.1797. In brief, β-lactamase sequences used in [129,134] were aligned in the MAFFT program under the E-Ins-I algorithm [160]. Evolutionary distances (in substitutions per site) were read from the branch lengths of a maximum likelihood tree constructed in the IQTree program [161] under the LG + G + I model selected by ModelFinder [162]; ultrafast bootstrap (UFBoot) with 1000 iterations and SH-aLRT branch test were used for branch support assessment, as implemented in IQTree [163,164]. There was no change in other parameter values we recently used [134]. Orange rectangles for cyclophilins do not clearly illustrate the association between increased evolutionary distances (CypA = 0.451 < CypB = 0.632 < CypC = 0.783) and higher overall dissipation, but Figure 3b resolves that issue. (**b**) The CypA < CypB < CypC ranking is clear here for the catalytic constant *k_cat_* and Dissipation/(*RT*) (both parameters are expressed in inverse seconds) [134], but enzyme efficiency (*k_cat_*/*K_M_*) ranking is different (blue points). All engineered glutamase racemase mutants [158] are regularly clustered together (red points) in the dissipation plane due to physical constraints. They exhibit higher dissipation and *k_cat_* values than the wild-type enzyme (green point).

While the log–log–log representation is effective for identifying scaling trends, it compresses relative distances and obscures evolutionary relationships within enzyme families. To address this limitation, Figure 3a plots evolutionary distances on the *y*-axis in a conventional three-dimensional space, with *k_cat_* on the *x*-axis and dissipation on the *z*-axis. For β-lactamases, the ranking PC1 < RTEM < Lac1 reproduces previous results [102], not only in terms of evolutionary distance but also for *k_cat_* and dissipation. Thus, more evolutionarily derived β-lactamases exhibit improved catalytic performance at the cost of increased housekeeping dissipation. A similar trend is observed for human cyclophilins, where the evolutionary ranking CypA < CypB < CypC coincides with increasing *k_cat_* values and dissipation, in agreement with our recent findings (Figure 6 in [134]).

Figure 3b further clarifies these relationships by replacing *k_cat_* with enzyme efficiency *k_cat_*/*K_M_* on the *x*-axis. Together, Figure 3a,b corroborate our phylogenetic analyses, showing that increasing evolutionary distance from the putative ancestral sequence is accompanied by systematic increases in dissipation and in the kinetic parameters *k_cat_* and *k_cat_*/*K_M_* [134]. Figure 3b also illustrates a rare but informative case in which engineered glutamate racemase variants—single-site (K29A, R214A, P99A, Y221A, K106A, R25A, Q86A) and double-site (R214A/K106A) mutants—exhibit higher turnover numbers and greater dissipation than the wild-type enzyme [30,134,158]. In the log–log–log representation of Figure 2, all glutamate racemase variants collapse onto an effectively single point on the dissipation-scaling plane. Figure 2 and Figure 3 together emphasize the dominance of physical constraints over evolutionary fine-tuning in determining scaling behavior.

### 6.3. Dissipation Landscape Constraints for Enzymes and Metabolism

Across all enzymes examined, encompassing four of the seven major EC classes and all three life domains (Bacteria, Archaea, and Eukarya, with 18 from *Homo sapiens*), dissipation exhibits a highly statistically significant correlation with both *k_cat_* and *k_cat_*/*K_M_* [134]. Generalist and specialized enzymes occupy the same dissipation plane, despite specialized enzymes displaying a median dissipation approximately 350-fold higher (Figure 4 in [134]). Even enzymes differing by nearly ten orders of magnitude in net forward flux—from carbonic anhydrase II and ketosteroid isomerase to glucose isomerase and 2-hydroxyisobutyryl-CoA mutase—remain constrained to this same plane. These observations demonstrate a synergy between physical (thermodynamic) constraints and biological evolution, which together define a dissipation landscape along which enzymes can evolve toward higher or lower performance. While biological context determines the evolutionary trajectory along the plane, physical laws restrict all enzymes—including engineered variants—to remain within it.

When expressed as a power law in the log–log representation of dissipation versus enzyme efficiency, the observed slope of approximately 0.72 implies that dissipation (or entropy production under isothermal conditions) scales as (*k_cat_*/*K_M_*)^0.72^. Intriguingly, Kleiber’s law [159] falls within the confidence interval of this slope, raising the question of whether sublinear scaling in enzyme catalysis may share a common physical origin with metabolic scaling at the organismal level, as discussed in [134]. Within the same confidence interval, *k_cat_* scales nearly linearly with dissipation (∼(*k_cat_*)^0.97^), as expected if the generalized flux–force product *JX* approximates the turnover number. However, this approximation holds only when backward fluxes are negligible. For the majority of the 75 enzymes analyzed, backward fluxes are significant, with ratios of *k_cat_*/(*J* = *J*_(+)_ − *J*_(−)_) exceeding 2.0 for 43 enzymes and reaching values above 100 for certain mutants, such as the D-psicose 3-epimerase R215K variant.

## 7. Discussion, Limitations, and Future Directions

This final section integrates discussion, limitations, and future directions, serving as a concluding synthesis of the review. We used the following scheme:

[Current limitations] → [Dissipation plane as unifying framework] → [Comparative synthesis] → [Future directions]

### 7.1. About the Association Between Enzyme Kinetic Parameters and Dissipation

A central theme of this review is the statistical association between enzyme catalytic performance and total entropy production. However, it is important to emphasize that increased catalytic rate does *not* necessarily imply increased dissipation for individual enzymes or enzyme variants. As discussed in detail in our recent quantitative analysis of kinetic datasets [134], the correlation between catalytic rate enhancement (e.g., *k_cat_* or *k_cat_*/*K*_M_) and dissipation emerges robustly only at the ensemble level when many enzymes spanning several orders of magnitude are analyzed together. When examined in linear (non–log-transformed) space, this association is considerably weaker, indicating that dissipation cannot be reliably predicted from catalytic rate alone. Thus, while a statistically robust positive correlation exists in log–log space, it should be interpreted as an emergent, population-level trend rather than a deterministic relationship applicable to individual enzymes [7].

The question of whether increased dissipation reflects operation further from thermodynamic equilibrium depends critically on how “distance from equilibrium” is defined. In some formulations, dissipation itself is used as a metric of nonequilibrium displacement, but such an approach risks circular reasoning and does not resolve whether dissipation increases *because* an enzyme operates further from equilibrium. For cyclic reaction schemes, displacement from equilibrium can alternatively be quantified by the thermodynamic force $X=ln\frac{\Gamma }{K_{eq}},\;$ where *Γ* = *c_p_*/*c_s_* is the mass action ratio, and *K_eq_* is the equilibrium constant, *K_eq_* = *c_p_*(*eq*)/*c_s_*(*eq*). This force determines the imbalance between forward and backward fluxes (*J*_(+)_ ≥ *J*_(−)_ for *X* ≥ 0) and provides a non-circular measure of thermodynamic driving [59,165].

Across the set of reactions analyzed here, positive force is invariably associated with positive entropy production. However, comparisons among individual isoenzymes reveal that increased dissipation does not necessarily correspond to increased thermodynamic force relative to wild-type enzymes. Indeed, while a weak power-law relationship between dissipation and force can be detected in log–log space, the large scatter (low R^2^) indicates that dissipation does not systematically increase simply because enzymes operate further from equilibrium [134]. Instead, dissipation reflects additional structural and dynamical features of the catalytic mechanism that are not reducible to driving force alone. Consistent with this interpretation, enzymes with widely different turnover numbers can exhibit similar dissipation levels, and conversely, dissipation can vary substantially while *k_cat_* remains nearly unchanged, underscoring that dissipation encodes mechanistic information beyond catalytic rate alone [7,166].

### 7.2. Regulated (Biology) and Constrained (Physics) Dissipation

Dissipation constraints are shared between physics, which sets the upper and lower bounds, and biology, which regulates dissipation within these bounds. The total dissipation spans many orders of magnitude across different enzymes, as do turnover numbers and catalytic activities. In this review, we presented evidence that physical and kinetic enzyme parameters are not independent. All are required for a meaningful characterization of enzyme functionality. This interdependence becomes apparent only when a complete set of microscopic kinetic constants is collected for a large number of enzymes, allowing the dissipation landscape—manifested as a dissipation-scaling plane—to emerge as a provisional pattern.

The biological context determines how enzymes from the same family evolve across this dissipation plane, whereas physical laws restrict all enzymes, including engineered variants, to remain within it. Physical (thermodynamic) evolution, long recognized in physics, is therefore not absent from biology. On the contrary, physical and biological evolution appear to be tightly coupled and often act synergistically. A defining capability of living systems is their ability to modulate the rates at which physical processes unfold, as reflected in changes in enzyme dissipation along the dissipation plane. For example, evolutionary pressure toward more efficient dissipation of free-energy gradients can, as a positive side effect, enhance catalytic performance. At this point, several clarifications regarding the interpretation of dissipation–performance relationships are warranted.

### 7.3. Dataset Qualifications

Several limitations currently restrict the universality of the proposed scale-invariant performance–dissipation relationships. Integral membrane proteins essential for bioenergetics [2] and oxidoreductases from EC class 1, the most populous enzyme class [167], are not yet represented in the enzyme datasets used to construct the dissipation-scaling plane. Also, we limited the dataset to mostly uni-uni enzymes that convert one substrate to one product, with all transition steps bidirectional and all microscopic rate constants observed or estimated for both directions. The justification for excluding some enzymes is the difficulty of determining the complete set of microscopic rate constants for complex kinetic schemes containing multiple cycles and irreversible steps, which are typical of oxidoreductases and free-energy-transducing membrane enzymes. A historical lack of interest in comprehensive kinetic characterization further limits dataset completeness. These challenges represent clear targets for future work aimed at expanding the bioinformatic foundation required for similar analyses. Moreover, defining appropriate in vivo substrate and product concentrations under homeostatic steady-state conditions remains non-trivial. It may be fundamentally incompatible with the requirement that all reactions proceed in the forward direction under near-physiological driving forces.

### 7.4. Comparative Synthesis

The present introduction of the dissipation plane as a unifying framework enables a comparative synthesis, as many previously published models of enzyme kinetics, thermodynamics, and evolution naturally occupy distinct regions of this plane. Table 1 summarizes canonical enzyme classes and theoretical models discussed in the literature and indicates their *qualitative* location on the dissipation plane. The placement reflects interpretive synthesis based on previously published kinetic and thermodynamic analyses and does not involve new experimental data, simulations, or parameter estimation. Molecular motors are included as a strongly non-equilibrium reference class to illustrate high-dissipation regimes relevant for biological function. Enzymes described as “perfect” are, for instance, triosephosphate isomerase [168,169], carbonic anhydrase [170], and ketosteroid isomerase [171]. In enzymology, “(catalytically) perfect” typically means the enzyme’s second-order catalytic efficiency *k_cat_*/*K*_M_ is so high that the overall rate is limited mainly by diffusion (how fast enzyme and substrate can find each other), rather than by chemistry at the active site.

Table 2 links long-standing evolutionary and functional questions in enzyme catalysis and biochemical networks to distinct qualitative regions of the dissipation plane. The cited studies do not explicitly employ the dissipation-plane framework; rather, they are mapped here to provide a unifying conceptual synthesis of how energetic dissipation constrains speed, accuracy, robustness, and evolutionary fitness.

Although the dissipation plane has been introduced here for the first time as an explicit conceptual framework, many classical and modern studies of enzyme kinetics, non-equilibrium thermodynamics, and molecular evolution implicitly explore restricted regions of this plane. Table 1 and Table 2 synthesize these contributions by mapping representative models, enzyme classes, and evolutionary questions onto qualitative dissipation regimes. This approach does not reinterpret existing results quantitatively, but instead highlights how disparate theoretical and experimental insights can be organized within a common thermodynamic–functional landscape. In this sense, the dissipation plane serves as a unifying lens rather than a replacement for established formalisms.

### 7.5. Future Directions

A more comprehensive dataset, combined with deeper integration of stochastic thermodynamics, will be required to assess the full generality of these findings. Notably, an alternative method for calculating total entropy production for a single enzyme (β-galactosidase) [181] yielded results consistent with both the present approach and our recent publication [151]. That study reached the same conclusion: increased enzyme efficiency is associated with higher total dissipation. Whether the correlated increases in evolutionary distance, catalytic performance, and housekeeping dissipation observed here extend broadly across enzyme families and throughout the history of life remains an open question. Figure 2 and Figure 3 provide only two representative examples—bacterial β-lactamases and human cyclophilins.

Nevertheless, recent advances in far-from-equilibrium thermodynamics applied to biological catalysis suggest that the dissipation-scaling plane may represent a generic organizing principle for enzyme evolution and design. Future research may either falsify or support this possibility and, critically, provide mechanistic explanations for the apparent emergence of order from diversity in enzyme kinetic and thermodynamic properties. One plausible mechanistic basis for dissipation–performance relationships is the directional motion of particles within the enzyme nanoworld triggered by substrate capture. Although thermodynamics alone cannot specify the identity of these particles, concerted proton transfer or proton shuttling through hydrogen-bonded networks frequently provides a compelling explanation for directional flux in enzymatic reactions.

Optimization of rate-limiting proton-transfer steps using extremal principles, such as variants of the maximum entropy production principle, represents one of several proposed routes by which dissipation may shape enzyme kinetics. To date, however, no authoritative comparative evaluation of such principles exists in enzymology or, more broadly, in the life sciences, leaving this question open for future investigation.

From a broader perspective, the dissipation-based framework discussed here should be viewed as complementary to established molecular and biochemical models of enzyme evolution rather than as an alternative to them. Mainstream evolutionary approaches have successfully elucidated how genetic variation, structural plasticity, catalytic promiscuity, and historical contingency shape enzyme function and innovation, and these processes remain central to our understanding of enzyme evolution [26,182]. The thermodynamic perspective adds an additional layer by highlighting how nonequilibrium constraints may delimit the accessible range of kinetic parameters within which such evolutionary processes operate [28]. In this sense, dissipation and entropy production do not replace conventional selective explanations but may help rationalize why certain catalytic regimes are repeatedly favored or avoided across divergent enzyme families. As quantitative links between kinetic parameters, dissipation, and evolutionary metrics continue to emerge, integrating thermodynamic constraints with molecular evolutionary frameworks may provide a more unified description of enzyme optimization in both natural evolution and enzyme engineering [28,183].

More generally, nonequilibrium physics provides numerous examples of ordered behavior emerging under sustained external forcing. In physical systems, order can arise when light interacts with chaotic Brownian dynamics [184]. Analogously, the conversion of ionic gradients into electrochemical energy through selective transport of Na^+^, K^+^, H^+^, and Cl^−^ may have played a central role in the formation of the first cells [185,186], representing another instance of complex order emerging from nonequilibrium constraints. Intensive free-energy conversion under far-from-equilibrium conditions is a defining asymmetry of living systems: life persists by continuously transducing environmental disequilibria into chemical work [185,187,188,189]. In the enzyme families discussed in this review, higher catalytic performance (e.g., catalytic efficiency and turnover) is, on average, associated with higher “housekeeping” dissipation, consistent with the idea that maintaining fast reaction cycles and functional order requires sustained entropy production.

A plausible inference is that, during abiogenesis, persistent abiotic driving forces were required to maintain comparably far-from-equilibrium conditions. In alkaline hydrothermal vent scenarios, natural pH and redox disequilibria across thin inorganic barriers could have generated proton-motive forces capable of powering early carbon fixation and energy conservation, before the evolution of genetically encoded ion pumps [190,191,192,193]. On this view, core features of bioenergetics—chemiosmotic coupling and vectorial biochemistry, including the selective transduction of ion gradients (H^+^, Na^+^, and, subsequently, other ions) into electrochemical energy—could predate the genetic code and may have scaffolded the emergence of the first cells [185,194].

On the whole, nonlinearity and sustained non-equilibrium forcing are widely recognized prerequisites for self-organization in complex chemical networks, including protometabolic systems that can develop autocatalytic feedbacks [8,195,196]. In that context, the dissipation–kinetics linkage emphasized in this review may reflect a deeper continuity: the same thermodynamic “capacity for dissipation” that supports accelerated reaction cycles in modern biochemistry could also have favoured the stepwise synthesis and stabilization of increasingly complex organic reaction networks during early evolution. Research at the interface of nonequilibrium physics and biology is therefore entering a particularly promising phase, with important implications for enzyme engineering, bioenergetics, evolutionary theory, origin-of-life research, and fundamental biophysics [197,198,199,200,201,202].

## Figures and Tables

**Table 1:** Representative enzyme and molecular machine models and their qualitative placement on the dissipation plane.

Enzyme/Model Class	Key Catalytic Feature	Dissipation Regime	Evolutionary Implication	References
Michaelis–Menten enzymes	Near-equilibrium catalysis	Low dissipation	Constrained efficiency	[169,172]
Metabolic flux enzymes	High throughput	Intermediate–high dissipation	Selection for speed	[173]
Molecular motors	Directional motion	Strongly non-equilibrium	Robust function under noise	[7,174]
Proofreading enzymes	High accuracy	High dissipation	Error suppression at energetic cost	[175,176]
“Perfect” enzymes	High catalytic efficiency	Very high dissipation (upper observed range)	Diffusion- controlled enzymes	[168,169,170,171]

**Table 2:** Conceptual correspondence between evolutionary questions and dissipation-plane regions.

Evolutionary Question	Relevant Plane Region	Literature Focus	Selected Literature
Speed vs. efficiency	Low dissipation edge	Enzyme kinetics	[169,170]
Robustness to noise	Broad high-drive regimes	Non-equilibrium models	[145,177,178]
Accuracy vs. cost	High dissipation region	Proofreading	[175,179]
Evolutionary fitness under thermodynamic constraints	Accessible region of the dissipation plane	Limits on selectable phenotypes and evolutionary pathways	[8,180]

## Data Availability

New evolutionary distances for β-lactamases and the methods used to derive them are briefly described in the legend to Figure 3a. These calculations mainly served to verify our older results available at https://github.com/DJureticSplit/PERF-ENZYMES (accessed on 31 January 2026). Otherwise, no new data were created or analyzed in this study. Data sharing does not apply to this article.

## Abbreviations and Symbols

The following abbreviations and symbols are used in this manuscript:

Abbreviation/Symbol	Definition
Φ	Dissipation function
*σ*	Local entropy production
*T*	Absolute temperature
*X_i_*	Generalized force for the process *i*
*J_i_*	Generalized flow for the process *i*
*μ*	Free energy
*S*	Entropy
$A_{i}$	Affinity for reaction *i*
EPR	Entropy production rate
MEPP	Maximum entropy production principle
MPEP	Maximal partial entropy production for a chosen *i* → *j* transition
*p_i_*	State *i* probability
*k_i_* or *k_ij_*	Microscopic kinetic constant for the transition *i* → *j*
$k_{i}^{*}$	Second-order rate constant (in M^−1^s^−1^ units) for enzyme-ligand association
*κ*	Parameter defining the angular position of the transition state
*k_cat_*	Catalytic constant (turnover number)
*K_M_*	Michaelis–Menten constant
*k_cat_*/*K_M_*	Enzyme efficiency (catalytic efficiency, specificity constant)
*K* or *K_eq_*	Equilibrium constant
E	Free enzyme
ES	Enzyme–substrate complex
EP	Enzyme–product complex
EZ	Intermediate complex
*c_s_* or [*S*]	Substrate concentration (mol)
*c_p_* or [*P*]	Product concentration (mol)
*Γ* = *c_p_*/*c_s_*	Mass–action ratio
*R*	Molar gas constant
CAII	Carbonic anhydrase II
KSI	Ketosteroid isomerase
PC1, RTEM, Lac1	Bacterial β-lactamases
CypA, CypB, CypC	Human cyclophilins
